# What will you want tomorrow? Children—But not adults- mis-predict another person’s future desires

**DOI:** 10.1371/journal.pone.0259159

**Published:** 2021-11-03

**Authors:** Gema Martin-Ordas, Cristina M. Atance

**Affiliations:** 1 Division of Psychology, University of Stirling, Stirling, United Kingdom; 2 School of Psychology, University of Ottawa, Ottawa, Canada; University of Queensland, AUSTRALIA

## Abstract

Young children have difficulty predicting a future physiological state that conflicts with their current state. This finding is explained by the fact that children are biased by their current state (e.g., thirsty and desiring water) and thus have difficulty imagining themselves in a different state (e.g., not thirsty and desiring pretzels) “tomorrow,” for example. Another potential explanation that we explore here is that young children have difficulty understanding how physiological states, like thirst, fluctuate over time. We asked 3-, 4- and 5-year-olds (Experiment 1) and adults (Experiment 2) to predict what a thirsty Experimenter–who preferred crisps to water—would want (“water” or “crisps”) “right now” and “tomorrow.” Only adults correctly predicted someone else’s future desires when this person’s future and current desires conflicted. In contrast, both adults and children in the control groups (in which the Experimenter was not thirsty) had no difficulty predicting that the Experimenter would want crisps “right now” and “tomorrow.” Our findings suggest that children’s difficulty predicting future desires cannot solely be attributed to their being biased by their current state since the children in our study were, themselves, not thirsty. We discuss our results in the context of children’s difficulty understanding fluctuations in physiological states.

## 1. Introduction

Correctly attributing mental states such as beliefs, knowledge, and desires to others allows us to function effectively in our social world [1]. Indeed, such attributions allow us to understand and predict other people’s actions and reactions. For example, knowing that your friend is afraid of dogs allows you to predict that she will not enjoy accompanying you while you walk your dog and, as such, it would not make sense to invite her to do so. Developmental research has shown that children’s abilities to attribute knowledge and beliefs to others improve between ages 3 and 5. For example, by age 4, children start to understand that the person who has looked inside a box will know its contents, whereas the person who has not looked inside will not [e.g., 2]. Similarly, around age 4, children begin to recognize that someone else’s mental states might differ from their own and also from reality or, so-called “false beliefs” [e.g., 3, 4].

Children’s reasoning about desires also improves substantially during early development. This understanding has most often been assessed using tasks that require children to acknowledge that although they, themselves, hold a particular desire—or preference- another individual may not. By 18 months of age, children show an emerging understanding that others’ preferences may differ from their own [e.g., 5] and, by 3 to 4 years of age, children begin to understand that another person’s desires may directly conflict with their own [e.g., 6–8]. Three-year-olds can also predict that someone who has had a positive experience with an object will probably want the object later, whereas someone who has had a negative experience with that same object will not—or, what has been termed “attitude-generated” desires [9]. In so doing, children demonstrate their understanding that desires can persist over time; that is, if someone likes something “now” they will probably continue to like it. Yet, an equally important aspect of desires is that they can *change* over time. Young children’s appreciation of this fact is the focus of the current study.

Understanding the changing nature of desires is vitally important to how we make decisions both for ourselves and others. For example, although we may have eaten a large meal and no longer desire food, or are particularly tired and have no desire to go for a jog, we nevertheless appreciate that these desires will change in the future. Although adults are by no means perfectly accurate in predicting such changes [e.g., 10], a complete failure to do so would result in empty kitchen cabinets, and multiple pairs of discarded running shoes. Similarly, appreciating that other people’s desires change with time allows us to act with their best interests in mind. For example, if I know that my friend is tired, I should not ask her to join me for a jog now; instead, I should wait and ask her tomorrow. In sum, understanding that desires change over time allows us to make effective decisions that are not solely based on our own and others’ current states but, importantly, on how we anticipate these states changing in the future.

When do young children begin to understand that what they or others desire now might be different from what was desired in the past, or what will be desired in the future? Gopnik and Slaughter [11] showed that by 4 years of age children were able to report a past desire even when this desire differed from their current one. For example, children ate a snack until they were no longer hungry. They were then asked whether they had been hungry *before* they ate the snack. Four-year-olds, but not 3-year-olds, correctly responded to this question by acknowledging their earlier physiological state of hunger [11].

Using a very different paradigm, Bélanger, Atance, Varghese, Nguyen, and Vendetti [12] assessed preschoolers’ ability to appreciate changes in their *future* or “grown-up” desires/preferences. Preschoolers were presented with a task in which they had to decide which of two items (e.g., Kool-Aid or coffee) they would prefer now and in the future (i.e., when they were “all grown up”). Results showed that children’s ability to understand that their current preferences (e.g., Kool-Aid) will differ from their future preferences (e.g., coffee) improves with age, and that only 5-year-olds can predict above chance what they will prefer as adults. Together, these two studies suggest that children’s understanding of changes in desires/preferences improves during the preschool years and that, by age 5, children appreciate that current desires may differ from past and future ones.

Interestingly, however, children as old as 7 struggle to predict “present to future” changes in desires stemming from *physiological* states, [13–16]—so-called “physiology-generated” desires [9]. For example, Atance and Meltzoff [13] designed a “Pretzel task” in which children were given pretzels to eat while the experimenter read them a story. Importantly, the purpose of asking children to eat pretzels was to induce thirst. Once the experimenter finished reading the story, one group of children was asked whether they would want water or pretzels “right now,” and one group was asked whether they would want water or pretzels “tomorrow.” Both groups overwhelmingly chose water (and did not differ in their tendency to do so) indicating that those children asked about “tomorrow” were unable to predict that their future preference would likely shift back to “pretzels.” These findings contrasted with those of the “right now” and “tomorrow” control conditions, in which children were not offered pretzels while reading the story, and were not thirsty at the time that they were asked about their present and future desires. Indeed, in these two conditions, children chose pretzels for “right now” and for “tomorrow.” Moreover, using the Pretzel task it has been shown that 8- to 13-year-olds and adults also experience difficulty accurately predicting future physiological states that conflict with their current ones or, what the authors term “induced-state episodic foresight” [17].

What might account for people’s difficulty making predictions about a future desire (e.g., “pretzels”) that conflicts with their current one (e.g., “water”)? The most prominent argument is that people’s current state *biases* their reasoning about their future state [e.g., 13–17]. That is, people’s current state of thirst–and, hence, desire for “water”—is highly salient thus making it difficult for them to step out of their current state, so to speak, to appreciate that they will no longer be thirsty (and thus desire pretzels) “tomorrow.” In fact, when Mahy [15] gave children water to drink after they had eaten pretzels, and then asked them once again to predict what they would prefer “tomorrow,” children shifted their preference to pretzels. This finding further supports the claim that children’s current state biases their predictions for the future. We hereafter refer to this as the “current state bias” account [cf., 16].

There is, however, another possible mechanism that could at least partly explain children’s poor performance in the Pretzel task: a difficulty appreciating the waxing and waning of physiological states, or what we term the “current state change” account [cf., 16]. More specifically, young children may simply fail to understand that their own level of thirst will decrease over time, thus leading them to prefer pretzels again the next day (although this possibility seems unlikely to account for adults’ failures, we nevertheless test a version of this hypothesis in Experiment 2 of the current study).

This hypothesis is supported by data from Moses et al. [9] in which 4- and 5-year-olds were told about two characters–one who had eaten a large amount of food (or drunk a large quantity of water) a long time ago, and another who had done so a short time ago. Strikingly, neither age group was proficient at understanding that only the character who had not eaten/drunk in a while would desire food/water. From these data, Moses et al. [9] concluded that preschoolers have a poor grasp of how experiences of satiation and deprivation lead to desire formation. Accordingly, their ability to reason about such physiology-generated desires is particularly difficult, and more difficult than reasoning about desires that arise from non-physiological states (i.e., attitude-generated desires). This may be because, for example, eating many salty pretzels generates an incongruent immediate desire—one becomes satiated and does not want to eat more pretzels. This kind of incongruence does not characterize attitude-generated desires—if one is enjoying playing with a dog, one usually wants to continue doing so.

Thus, in the context of the Pretzel task, children may not fully appreciate that although they are currently thirsty and desire water “now,” thirst will wane over time and a desire for pretzels will likely return tomorrow. Importantly, failure to appreciate this critical fact about physiology-generated desires would lead children to err not only when making predictions about their own future desires—as previous studies have shown [e.g., 13]- but also when predicting the future desires of others. This is a critical point because by the current state bias account, children should have little or no difficulty predicting that someone else who is thirsty would desire pretzels (and not water, “tomorrow”). This is because children, themselves, would not be biased by their own current state of thirst.

Our goal in Experiment 1 was to examine whether children’s mis-predictions about future desires might be explained by a failure to understand that the physiological states underlying such desires change over time (i.e., current state change account). To do so, we adapted the Pretzel task previously developed by Atance and Meltzoff [13] to test whether 3-, 4- and 5-year-old children could accurately predict *someone else’s* current and future desires. In Experiment 2 we further examined the current state change account by presenting adults with the same task that children received in Experiment 1.

## 2. Experiment 1

Recently, Mazachowsky et al. [16] presented 3- to 7-year-old children with the Pretzel task [13]. Once children had consumed pretzels and became thirsty, they were asked to decide both what they, themselves, and also a thirsty experimenter—who preferred pretzels over water- wanted to have “tomorrow”, pretzels or water. Children were significantly more likely to correctly predict the Experimenter’s future preference (i.e., pretzels) compared with themselves (supporting the “current state bias” account). However, for neither “self” nor “other” did children choose pretzels significantly more than chance (also supporting the “current state change” account). Part of the difficulty disentangling these two accounts is because in Mazachowsky et al.’s design, children, themselves, were thirsty. What is thus needed is a design in which children themselves are not thirsty but must make choices for “now” and “tomorrow” for a thirsty or non-thirsty experimenter.

Accordingly, in our study, preschoolers were presented with an adapted version of Atance and Meltzoff’s [13] Pretzel task. Children were told that an adult had eaten a bag of salted crisps (or “chips” in North America), and consequently, that she was very thirsty. After a short story was read to them by the Experimenter, children were asked to predict whether the Experimenter would want to have “crisps” or “water” either “right now” or “tomorrow.” Importantly, children were also told that the Experimenter preferred crisps to water. We also asked children to explain their choices for the Experimenter. Importantly, only the Experimenter’s–and not the child’s—current state was manipulated; a critical difference between our method and Mazachowsky et al.’s [16].

If, as previous researchers have argued, children’s difficulty accurately predicting future physiological states that conflict with current states is due to current state bias, then (non-thirsty) children as young as 4 or 5 years of age should have little, or no, difficulty making a prediction about *another* person’s physiology-generated desire. This is because children, themselves, are not biased by their current state and because, by ages 4 and 5, children appear to have a general understanding that desires change over time [e.g., 11, 12]. On the other hand, if children’s difficulty is largely rooted in mis-understanding the waxing and waning of physiological states, then they should have difficulty predicting that a thirsty Experimenter will desire their preferred food (i.e., crisps) tomorrow.

### 2.1. Methods

#### 2.1.1. Participants

Participants were 147 typically-developing children; three were excluded due to experimenter error, resulting in a final sample of 144 children (76 females; 68 males) aged 3 (*M* = 42 months, *Range* = 36 to 47, *n* = 49), 4 (*M* = 52 months, *Range* = 48 to 58 *n* = 48), and 5 (*M* = 66 months, *Range* = 60 to 71, *n* = 47). See Table 1 for the sample broken down by age, gender and condition. Participants were predominantly White, middle class, and fluent in English and were tested individually in a quiet area of the Centre for Life, at the University of Newcastle or in the child lab facilities of the Institute of Neuroscience. Our experiment received ethical approval from the University’s Faculty of Medical Sciences Ethics Committee (project “The development of Future Thinking”). Parents provided written informed consent for their children’s participation, and children also provided their verbal assent.

**Table 1:** Number of participants per experimental condition as a function of age and gender.

	Choice for “right now”		Choice for “tomorrow”	
	Experimenter thirsty	Experimenter non-thirsty	Experimenter thirsty	Experimenter non-thirsty
**3YO**	M = 7; F = 6	M = 6; F = 6	M = 5; F = 6	M = 4; F = 9
**4YO**	M = 8; F = 2	M = 6; F = 8	M = 2; F = 11	M = 8; F = 3
**5YO**	M = 7; F = 4	M = 3; F = 9	M = 4; F = 9	M = 6; F = 5
**Adults**	M = 5; F = 7	M = 4; F = 8	M = 3; F = 9	M = 2; F = 10

#### 2.1.2. Materials and procedure

We used two full bags of salted crisps (25 g each), an empty bag of salted crisps, two bottles of water (330 ml each), and two storybooks. A picture of a child going to bed and waking up in the morning was used to explain the concept of “tomorrow.” We adapted Atance and Meltzoff’s [13] Pretzel task to include four conditions defined by the Experimenter’s (i.e., other’s) current state (“thirsty” or “not thirsty”), and the temporal marker corresponding to whether the choice that children needed to make was for the Experimenter “right now” or “tomorrow”:

*2*.*1*.*2*.*1*. *Experimenter thirsty–choice for “right now*.*”* Children were told that right before they had arrived, the Experimenter (E) had eaten a bag of salted crisps—E pointed to an empty bag of crisps- and stated “…*right now I am very thirsty*.” E then explained that because she “*had some crisps to eat*, *I am not hungry*.” Finally, E also stated *“And you know what*? *I like crisps more than water*.” At this point, E asked the child (1) “*Are you hungry right now*?”, (2) “*Are you thirsty right now*?”, and (3) “*What do you like best*: *crisps or water*?” The order in which questions (1) and (2) were asked was counterbalanced across participants. The rationale behind these three questions was to obtain a measure of children’s current state, as well as their preference for crisps vs. water. Next, E and child read a storybook for approximately 3 min. E then put away the storybook, showed the child a second storybook, and said “*Imagine that we are going to read this book right now*.” Next, E placed two bags of crisps and two bottles of water on the table and asked the two following questions: (1) “Self” question: “*What would you like to have while we read the book right now*? *Some crisps to eat or some water to drink*?” Once the child answered this question, E proceeded to ask why s/he made that choice (i.e., *“how come you chose that*?*”*). This question was only asked once, and if children failed to respond the E proceeded to ask the next question. (2) “Other” question: “*What do you think I would like to have while we read the book right now*? *Some crisps to eat or some water to drink*?” Once the child answered the “other” question, E proceeded to ask why s/he made that choice (i.e., *“how come you chose that*?*”*). The order in which the “Self” and “Other” questions were asked was counterbalanced for each of the four conditions. Importantly, the order of these questions did not affect children’s choices for the E (*χ*^2^ = .00, *df* = 1, *p* = .991) or for themselves (*χ*^2^ = 0.33, *df* = 1, *p* = .561) for either the “right now” or “tomorrow” conditions.

*2*.*1*.*2*.*2*. *Experimenter thirsty–choice for “tomorrow*.*”* We used the same procedure as for the “Experimenter thirsty–choice for right now” condition until right after the reading of the storybook. At this point the E said to the child “*Imagine that we are going to read this book tomorrow*.” E explained the concept of “tomorrow” by showing the child a timeline with a picture of a little child going to bed and waking up in the morning. The E then said “*everything that happens when he wakes up in the morning is tomorrow*.” The “Self” and “Other” questions were then as follows: “*What would you like to have while we read the book tomorrow*? *Some crisps to eat or some water to drink*?” and “*What do you think I would like to have while we read the book tomorrow*? *Some crisps to eat or some water to drink*?”

*2*.*1*.*2*.*3*. *Experimenter not thirsty–choice for “right now*.*”* This condition was identical to the “Experimenter thirsty- choice for right now” condition, save that children were not told that E had eaten a bag of crisps, or that E was thirsty, and not hungry. Importantly, however, as in the “Experimenter thirsty- choice for right now” condition, the E told children that she liked crisps more than water.

*2*.*1*.*2*.*4*. *Experimenter not thirsty–choice for “tomorrow*.*”* This condition was identical to the “Experimenter thirsty- choice for tomorrow” condition, save that children were not told that E ate a bag of crisps, or that E was thirsty, and not hungry. As in the “Experimenter thirsty- choice for tomorrow” condition, E told children that she liked “crisps” more than “water” and also explained to the children the concept of “tomorrow.”

The rationale behind the two “Experimenter not thirsty” conditions was to ensure that children would choose E’s preferred food item (i.e., “crisps”) for “right now” or for “tomorrow” when E made no statements about thirst or hunger. This allowed us to assess whether our experimental manipulation was successful. Children were randomly assigned to each of the four conditions.

#### 2.1.3. Scoring and analyses

Sessions were video-recorded and we scored children’s choices for the “Self” and “Other” questions (crisps = 1; water = 0). Children’s responses about their current states were coded as “1” if they reported to be hungry or thirsty and “0” if they said that they were not hungry or thirsty. We also coded children’s explanations for their choices for the E into the following categories: (1) E’s current state (e.g., “because you are thirsty”), (2) E’s future state (e.g., “because you might be thirsty”), (3) child’s current and future state (e.g., “because I am thirsty”, “because I might be thirsty”), (4) general preference (e.g., “because you like crisps”), and (5) no response/“don’t know”/irrelevant/general statement (e.g., “because you are a girl”, “because water is healthy”). See Explanatory note for an explanation on why these explanations were not analyzed. A second coder blind to the hypotheses coded 20% of the trials and reliability was 100% for children’s choices (i.e., “water” or “crisps”).

We used Pearson chi-square tests to analyze children’s performance in the four conditions for the “Self” and “Other” questions, and to analyze the effect of age. Phi (*φ*) was used to calculate effect sizes. We used multiple logistic regression tests to analyze the effect of children’s current states (i.e., whether or not they were thirsty and whether or not they were hungry) on their choices for the E, and binomial tests to assess whether children were above chance in the “Self” and “Other” questions (chance = 50%). All statistical tests were two-tailed and results were considered significant if *p* < .05.

### 2.2. Results

#### 2.2.1. “Other” questions

**Fig 1:**
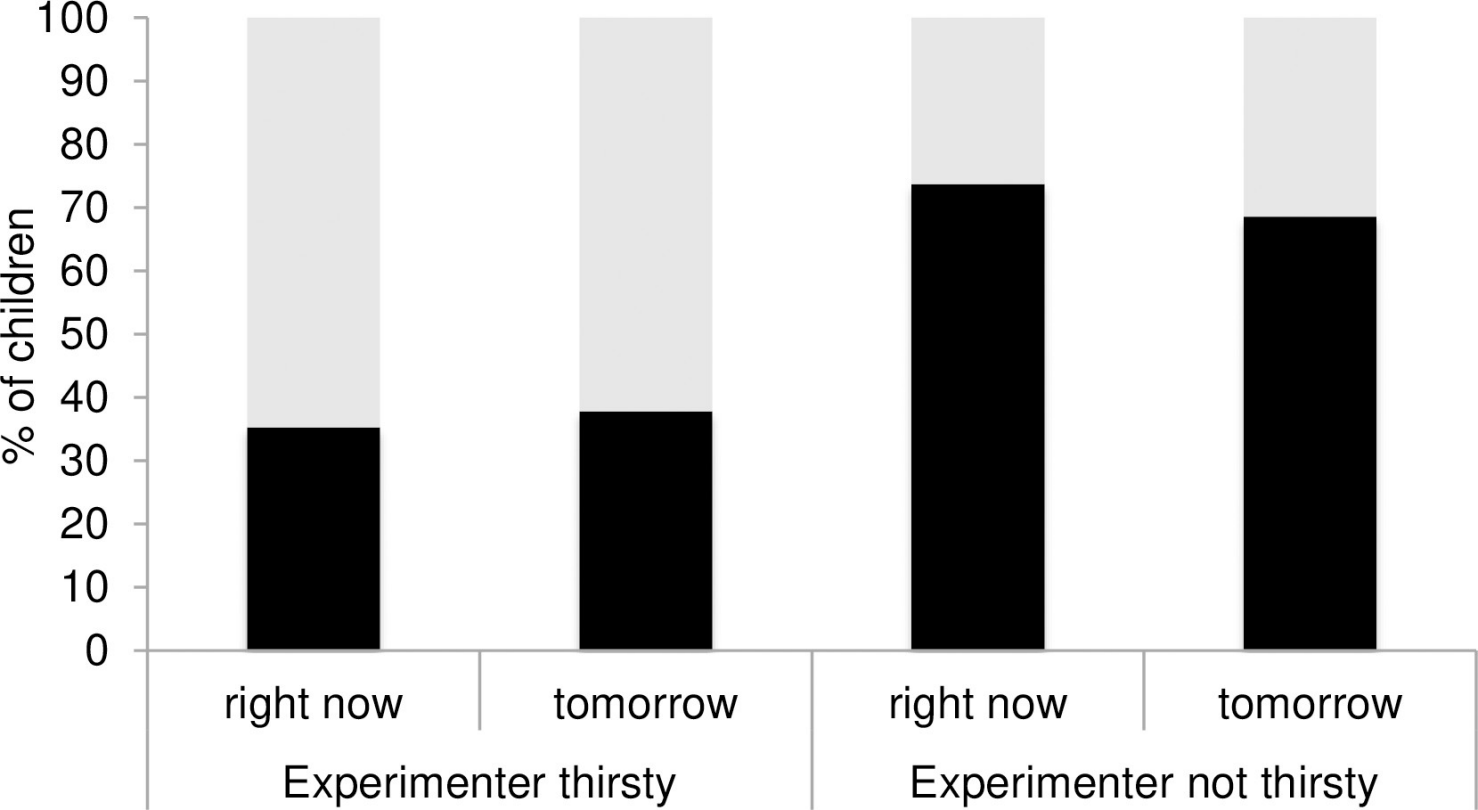
Percentage of children choosing crisps (in black) and water (in grey) for the experimenter in the “right now” and “tomorrow” conditions as a function of the experimenter’s current state (e.g., thirsty, not thirsty).

Consistent with the current state change account, children chose “water” just as often for the thirsty E when the choice was for “now” as when the choice was for “tomorrow” (*χ*^2^ = .049, *df* = 1, *p* = .824). Moreover, children chose “crisps” more often in the “Experimenter not thirsty-choice for tomorrow” condition than in the “Experimenter thirsty-choice for tomorrow” condition (*χ*^2^ = 6.81, *df* = 1, *p* = .009, *φ* = -.29), and this pattern did not change with age (*χ*^2^ = .78, *df* = 2, *p* = .677). As expected, children chose “crisps” for the E more often in the “Experimenter not thirsty-choice for right now” condition than in the “Experimenter thirsty-choice for right now” condition (*χ*^2^ = 10.71, *df* = 1, *p* = .001, *φ* = -.38), and this pattern did not differ as a function of age (*χ*^2^ = .83, *df* = 2, *p* = .660) (see Table 2 for data broken down by age and condition). Finally, children chose “water” for the E significantly above chance in the “thirsty” conditions (binomial test: *p* = .032) (see Fig 1).

**Table 2:** Percentage of participants choosing crisps for the experimenter as a function of age and condition.

	Choice for “right now”		Choice for “tomorrow”	
	Experimenter thirsty	Experimenter non-thirsty	Experimenter thirsty	Experimenter non-thirsty
**3YO**	38	83	36	76
**4YO**	40	71	38	54
**5YO**	27	67	38	72
**Adults**	17	83	92	83

As expected, when the E was not thirsty, children had no difficulty selecting “crisps” for her both for “now” and “tomorrow” (binomial tests: *p* = .005, and *p* = .041, for the “Experimenter not thirsty-now,” and “Experimenter not thirsty-tomorrow” conditions, respectively).

#### 2.2.2. “Self” questions

Children’s choice of “crisps” did not differ between the “Experimenter not thirsty-choice for right now” and the “Experimenter thirsty-choice for right now” conditions (*χ*^2^ = .68, *df* = 1, *p* = .409) and did not change with age (*χ*^2^ = .587, *df* = 2, *p* = .745). The same was true for the “tomorrow” conditions (*χ*^2^ = .73, *df* = 2, *p* = .786) (see Table 3 for data broken down by age and condition). Overall, children chose “crisps” for themselves significantly above chance in the “Experimenter thirsty” conditions—independently of the temporal marker- (binomial test: *p* = .017) and “Experimenter not thirsty” conditions (binomial test: *p* = .003). In other words, the E’s current state did not have an impact on which item children chose for themselves.

**Table 3:** Percentage of participants choosing crisps for themselves as a function of age and condition.

	Choice for “right now”		Choice for “tomorrow”	
	Experimenter thirsty	Experimenter non-thirsty	Experimenter thirsty	Experimenter non-thirsty
**3YO**	69	58	81	83
**4YO**	50	85	46	36
**5YO**	72	75	69	63
**Adults**	33	25	25	83

#### 2.2.3. Children’s current states

Children’s current states (e.g., hunger, thirst) did not affect their choices for the E when the E was thirsty. This was true for both the “right now” and “tomorrow” conditions (hunger: *Wald* = 1.35, *SE* = .53, *p* = .245; *Odds ratio*: 1.86; thirst: *Wald* = 2.33, *SE* = .55, *p* = .127, *Odds ratio*: .42). Children’s current state of hunger did however affect their choice of “crisps” for the E in the “Experimenter not thirsty” conditions, (*Wald* = 5.09, *SE* = .61, *p* = .024; *Odds ratio*: 3.99), whereas their current state of thirst did not (*Wald* = 1.58, *SE* = .63, *p* = .207; *Odds ratio*: .45). In other words, when children were hungry, they tended to more often choose “crisps” for the E as compared to when they were not.

### 2.3. Discussion

The results from Experiment 1 strongly suggest that part of children’s difficulty predicting future desires (that conflict with current ones) lies in their failure to understand fluctuations–or the “waxing and waning”–of physiological states. Indeed, although preschoolers accurately used the Experimenter’s current state (i.e., thirst) to predict what she desired “right now”, the Experimenter’s current state (i.e., thirst) appeared to bias children’s predictions about what she would desire in the *future*. This occurred despite the Experimenter explicitly telling children that she preferred “crisps” to “water.”

These data thus provide strong support for the current state change account given that *non-thirsty* children struggled to decide that a thirsty experimenter would prefer to have their favourite snack tomorrow. Importantly, children’s own food preferences (i.e., “crisps”) or current states (i.e., thirst, hunger) did not affect their choices for the Experimenter when she was thirsty, though children’s reported current state of hunger seemed to have an effect on their choices for the Experimenter when she did not mention being hungry or thirsty (i.e., “Experimenter not thirsty” conditions). We will return to this issue in the General Discussion. Similar to previous findings using the Pretzel task [e.g., 13–16], children’s performance did not improve with age: Five-year-olds were just as likely as 3-year-olds to incorrectly predict that the thirsty person would still want “water”–rather than her preferred option, “crisps”—the next day.

Although our results offer support for the current state change account, one could argue that children’s (especially younger ones) difficulty in our task was due to their mis-understanding the concept of “tomorrow.” For example, previous research has shown that it is only by the age of 5 that children start to have an understanding of “tomorrow” [e.g., 18–23]. We did, however, present children with a timeline in which “tomorrow” was physically represented, as well as conceptually explained. Nonetheless, an intriguing possibility is that different cognitive mechanisms underlie younger and older children’s failures in our task. More specifically, whereas a lack of understanding of how physiological states change over time as well as a lack of a mature concept of “tomorrow” may account for younger children’s failures, a more specific failure to understand the waxing and waning of physiological states might characterize the older children’s.

Recently, Kramer et al. [17] showed that adults experience as much difficulty as children when predicting future desires that conflict with their current ones. Thus, testing adults on the current paradigm should help to shed light on the mechanism underlying children’s performance (i.e., current state change account vs current state bias account). This is because adults should have a better sense of the former (i.e., how physiological states change with time) than young children but, as the research we have just described suggests, are still limited by the latter (i.e., current state bias). Accordingly, if our task is challenging for children because it requires that they understand the waning of a physiological state such as thirst, then we would expect adults to perform significantly better than children. Thus, in Experiment 2, we examined this possibility by presenting adults with the same task that children received in Experiment 1.

## 3. Experiment 2

As in Experiment 1, adult participants were told that the Experimenter had eaten a bag of salted crisps, and consequently, that she was very thirsty. After reading a newspaper for 3 min, participants were asked to predict what the Experimenter would want (i.e., “water” or “crisps”) either “right now” or “tomorrow” and to explain their item choices. As in Experiment 1, only the Experimenter’s current state was manipulated.

### 3.1. Methods

#### 3.1.1. Participants

Forty-eight young adults (34 females; 14 males) aged between 18 and 35 years participated in the study. See Table 1 for sample broken down by gender and condition. Participants were recruited using the University of Newcastle database. The ethnic composition of the sample was 95% White and 5% Asian, and all participants were predominantly middle class, and fluent in English. The experiment received ethical approval from the University of Newcastle’s Faculty of Medical Sciences Ethics Committee (project “Future Thinking in Adults”). Adult participants provided written informed consent and they were tested individually in the lab facilities of the Institute of Neuroscience.

#### 3.1.2. Material and procedure

We used the same materials as in Experiment 1. We also followed the same procedure as in Experiment 1, save for the following two differences: (1) Adults were not explained the concept of “tomorrow”; (2) the E and participant did not read a storybook—instead, E provided participants with a newspaper to read for 3 min. All sessions were video-recorded.

#### 3.1.3. Scoring and analyses

Adult participants’ responses were scored and analyzed in the exact same way as the children’s. A second coder blind to the hypotheses coded 20% of the explanations. Reliability was 100% for participants’ choices (i.e., “water” or “crisps”), for the verbatim explanations, and for the categorization of the explanations.

### 3.2. Results

#### 3.2.1. “Other” questions

**Fig 2:**
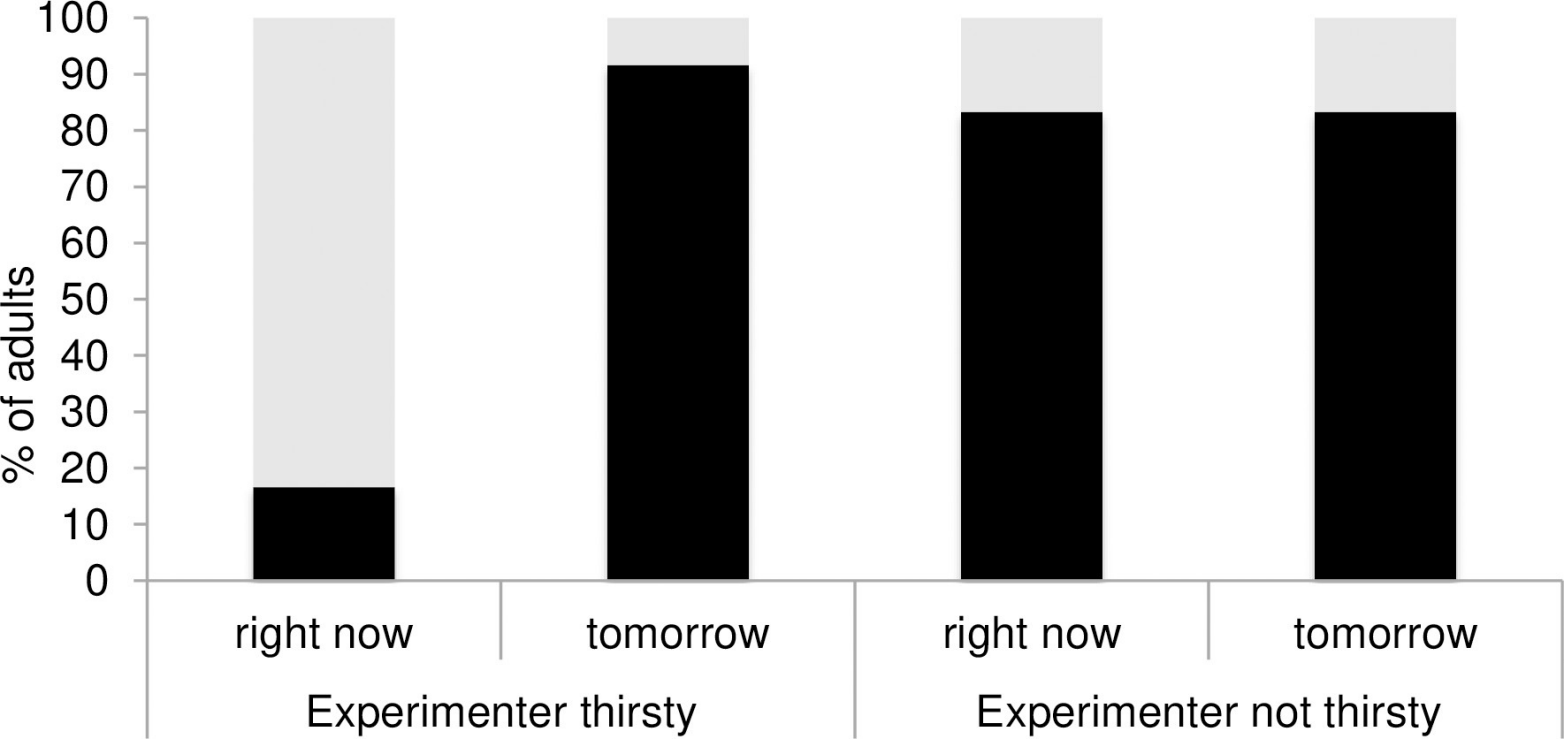
Percentage of adult participants choosing crisps (in black) and water (in grey) for the experimenter in the “right now” and “tomorrow” conditions as a function of the experimenter’s current state (e.g., thirsty, not thirsty).

Adults chose “water” more often for the thirsty E when the choice was for “right now” than when the choice was for “tomorrow” (*χ*^2^ = 13.59, *df* = 1, *p* < .001, *φ* = -.75). Moreover, adults chose “crisps” as often in the “Experimenter not thirsty-choice for tomorrow” condition as in the “Experimenter thirsty-choice for tomorrow” condition (*χ*^2^ = .38, *df* = 1, *p* = .537). As expected, adults chose “crisps” for the E more often in the “Experimenter not thirsty-choice for right now” condition than in the “Experimenter thirsty-choice for right now” condition (*χ*^2^ = 10.66, *df* = 1, *p* = .001, *φ* = -.66). Finally, adults chose “water” for the E significantly above chance in the “thirsty” condition when the choice was for “now” (binomial test: *p* = .039) and “crisps” when the choice was for “tomorrow” (binomial test: *p* = .006) (see Fig 2). As expected, when the E was not thirsty, adults selected “crisps” for the E both for “now” and “tomorrow” (binomial tests: *p* = .039 in both cases).

#### 3.2.2. “Self” questions

Overall 43% of the adults chose “crisps” as their preferred item and the remaining 57% chose “water.” Adults’ choices of “crisps” did not differ between the “Experimenter not thirsty-choice for right now” and the “Experimenter thirsty-choice for right now” conditions (*χ*^2^ = 0.20, *df* = 1, *p* = .653). However, adults chose “crisps” for themselves more often in the “Experimenter not thirsty-choice for tomorrow” condition than in the “Experimenter thirsty-choice for tomorrow” condition (*χ*^2^ = 8.22, *df* = 2, *p* = .004, *φ* = .54). Overall, adults did not choose “crisps” for themselves significantly above chance in the “Experimenter thirsty” conditions—independently of the temporal marker- (binomial test: *p* = .064) or in the “Experimenter not thirsty” conditions (binomial test: *p* = .833). In other words, the E’s current state did not have a clear impact on which item adults chose for themselves.

#### 3.2.3. Adults’ current states

Adults’ current states (e.g., hunger, thirst) did not affect their choices for the E when the E was thirsty. This was true for both the “right now” and “tomorrow” conditions (hunger: *Wald* = 4.37, *SE* = 1.00, *p* = .500; *Odds ratio*: .51; thirst: *Wald* = .22, *SE* = .89, *p* = 0.887, *Odds ratio*:1.14). The same was true when the E was not thirsty—for both “right” and “tomorrow” (hunger: *Wald* = 3.89, *SE* = 1.26, *p* = .532; *Odds ratio*: .45; thirst: *Wald* = .40, *SE* = 1.12, *p* = 0.520, *Odds ratio*: 2.03).

#### 3.2.4. Adults’ explanations

We asked adults to explain their item choices to determine whether they referred to the Experimenter’s current/future state, their own current/future state, or general statements (see Table 4 for the frequency of adults’ explanations in each of the categories). Responses from category 5 (i.e., “no response”) were not included in the analyses. The results showed that adults’ explanations were more likely to refer to the Experimenter’s current state in the “Experimenter thirsty-choice for right now” condition than in the “Experimenter not thirsty-choice for right now” condition (*χ*^2^ = 19.35, *df* = 2, *p <* .001, *φ* = .91). However, the type of explanation that adults provided did not differ between the “Experimenter thirsty- choice for tomorrow” and the “Experimenter not thirsty-choice for tomorrow” conditions (*χ*^2^ = 4.00, *df* = 3, *p* = .478). Note, that in both conditions most of the explanations referred to the Experimenter’s general preferences (see Table 4). Finally, adults’ explanations were more likely to refer to the Experimenter’s current state in the “Experimenter thirsty-choice for right now” condition and to the Experimenter’s general preference in the “Experimenter thirsty-choice for tomorrow” condition (*χ*^2^ = 12.67, *df* = 1, *p* = .001, *φ* = -.74).

**Table 4:** Adults’ explanations for the experimenter for the “choice right now” and “choice for tomorrow” by condition.

	Choice for “Right now”		Choice for “tomorrow”	
	Experimenter thirsty	Experimenter non-thirsty	Experimenter thirsty	Experimenter non-thirsty
**Experimenter’s current state (1)**	10	0	2	0
**Experimenter’s future state (2)**	0	0	0	1
**Participant’s current or future state (3)**	0	0	0	0
**General preference (4)**	1	10	10	10
**No response/don’t know/irrelevant (5)**	0	2	0	1
**Total**	**11**	**12**	**12**	**12**

### 3.3. Discussion

Adults correctly predicted both the Experimenter’s current desire and, importantly, this person’s future desire that conflicted with her current one; that is, adults predicted that the thirsty Experimenter desired water “right now” and her favorite snack (i.e., crisps) “tomorrow.” In addition, participants’ explanations took into account the Experimenter’s current state (i.e., thirst) to predict what she desired “right now” and the Experimenter’s general preferences (i.e., crisps) to predict what she would desire “tomorrow.”

Similar to the findings with children, adults’ own item preferences or current states (i.e., thirst, hunger) did not affect their choices for the Experimenter. However, we found that participants chose crisps for themselves more often in the “Experimenter not thirsty” condition compared to the “Experimenter thirsty” condition. Although one could argue that the Experimenter’s current state biased participants’ choices for themselves in the future, by this account participants should also have chosen water for “right now” more often in the “Experimenter thirsty” condition compared to the “Experimenter not thirsty” condition. Yet, this is not what we found. Alternatively, participants might have anticipated their state of hunger when returning to the lab “tomorrow” and, hence, chosen the crisps. If this were true, one would have also expected them to choose crisps in the “tomorrow” condition when the Experimenter was thirsty, which we did not find. Thus, given the inconclusive nature of this finding, future replication is warranted.

Importantly the results of this experiment help to differentiate between the current state change and the current state bias accounts. As predicted, adults did not have difficulty anticipating that the thirsty experimenter would desire crisps “tomorrow”—indicating that they were able to anticipate changes in the Experimenter’s desires between “right now” and “tomorrow.” This finding strengthens the claim that our task is challenging for children because it requires that they understand the waning of physiological states.

## 4. General discussion

Preschoolers and adults were presented with an adapted version of the Pretzel task [13] in which someone else’s current physiological state was manipulated. Participants were asked to predict what the other person desired “right now” and “tomorrow.” Our results showed that only adult participants correctly predicted someone else’s future desires when this person’s future (e.g., wanting to eat crisps) and current (e.g., wanting to drink water) desires conflicted. More specifically, Experiment 1 showed that preschool children correctly predicted what the thirsty Experimenter desired “right now” (i.e., water), but mis-predicted what she would desire “tomorrow” (i.e., crisps). Adult participants, on the other hand, correctly predicted what the thirsty Experimenter desired “right now” and “tomorrow.” Adults also referred to the Experimenter’ s current state when explaining their choices for “right now” and to the Experimenter’s general preferences when explaining their choices for “tomorrow.” Finally, when the Experimenter was not thirsty, both children and adults successfully predicted the Experimenter’s current and future desires (i.e., crisps in both cases).

Children and adults took the Experimenter’s current state of thirst into account when making predictions for the Experimenter “right now.” Recall that participants, themselves, were not in a current state of thirst when making predictions for the Experimenter. As such, these data show that children whose states have not been manipulated can readily predict someone else’s current desire, even when this desire does not fully align with their own. Indeed, the fact that children were significantly above chance in all three conditions save for the “Experimenter thirsty–choice for tomorrow” condition is important because it suggests that children understood the wording of the task and were not simply responding randomly.

Children’s choices for the Experimenter in the “Experimenter not thirsty” conditions were somewhat affected by children’s reported current state of hunger which suggests that children’s current state may have had some impact on their decision-making–especially when another person is not experiencing a salient physiological state, such as thirst. However, it is more likely that children were taking the Experimenter’s food preferences into account to predict what she would like to have “right now” and “tomorrow.” Otherwise, children’s reported current state of thirst would have affected their choices for the Experimenter and this is not what we found. Moreover, the fact that children predicted that the thirsty Experimenter would want water “right now” and “tomorrow” demonstrates that children were taking the Experimenter’s *current state* (which differed from the child’s own) into account. Why then did preschoolers fail to predict that the experimenter would want crisps *tomorrow*?

The results from Experiment 1 suggest that understanding desires that stem from physiological states is particularly difficult for preschool children [9, 15]. Indeed, had children understood how physiological states change over time, we would have expected them to base their predictions in the “Experimenter thirsty-choice for tomorrow” condition on the Experimenter’s general preferences (e.g., crisps)—rather than on the Experimenter’s current state of thirst. In fact, this is what adults did; that is, they took into account the Experimenter’s current state of thirst when predicting what she wanted “right now” and her general preferences when predicting what she wanted “tomorrow.”

It may be that a fully-developed understanding of physiological states (e.g., thirst) entails not only appreciating that such states change over time, but also that these changes are dependent on particular behaviors (i.e., drinking water). For example, if the thirsty Experimenter fails to satiate her thirst between “right now” and “tomorrow”, she will still be thirsty tomorrow and, consequently, will desire water. Because our participants were not given information about what would happen between “right now” and “tomorrow” (consistent with how this task has been administered in the “self” version, e.g., 13) one could argue that children, at least, made their predictions based on the assumption that the Experimenter would remain thirsty until “tomorrow.” Yet, given that even very young children have well-developed script-based knowledge about how routine events unfold [e.g., 24], it is unlikely that they do not have the knowledge that some “drinking” will happen in a 24-hr period (e.g., at mealtimes, etc.).

Moreover, what is especially striking is that there is *no* difference in performance between the *Experimenter thirsty–choice for “tomorrow”* condition and the *Experimenter thirsty–choice for “right now”* condition. That is, although it is plausible that some children might fail to take into account that the Experimenter will drink between now and tomorrow, it seems unlikely that all of them would. Accordingly, one would have expected some difference between the two “thirsty” conditions. In contrast, if *drawing* on the knowledge that drinking will happen between now and tomorrow when making their decisions is what is difficult for children then the pattern of results we obtained makes sense. This then suggests that a mature understanding of physiological states entails a two-step process in which one must first consider the possibility that particular behaviors (e.g., drinking water) will take place between “right now” and “tomorrow” and then, afterwards, incorporate this information into the decision-making process. If so, then we would predict that explicitly telling children that the Experimenter will drink between now and tomorrow would improve their performance. However, giving children this information would also fundamentally change the nature of our task and also diverge from how “self” versions of this task have been administered [e.g., 13–17]. That is, participants in self versions of the task are not told/reminded that they will be drinking between now and tomorrow. As such, an intriguing possibility is that giving participants this information may in fact improve performance (for both children and adults alike). Future task versions incorporating this addition would thus be worthwhile to run because they might shed further light on children’s and adults’ reasoning processes in such tasks.

Finally, an alternative possibility for children’s failure on our task is that they incorporated the crisp-eating event into their script for what will happen “tomorrow.” This is because in the only interaction that children had with the Experimenter, she ate crisps before meeting them. However, by this account, *older* children and adults, whom we’d expect to have stronger script-based knowledge [24] should have made more errors on our task, which is not what we found.

Interestingly, the fact that adults passed our “other” version of the task but not the “self” version in Kramer et al.’s [17] study suggests that two-step process involved in the understanding of physiological states may be more complex when it is the “self” who is experiencing the visceral state (e.g., thirst) in question. One possibility is that when adults find themselves in a salient current state, such as thirst, their focus rests on how to address this state “now”, with little cognitive resources devoted to reasoning through how a future action (i.e., drinking) would alleviate this state. Future studies should address this issue by directly comparing participants’ performance on “Self” vs. “Other” versions of this task in which both the participants’ and the Experimenter’s current states are/are not manipulated and participants are/are not given explicit information that they will have a drink between “now” and “tomorrow.” These comparisons would shed further light on the kinds of contexts that are particularly challenging for young children’s (and adults’) future-oriented decision-making, both for themselves and for others.

In conclusion, our study shows that whereas adults successfully predicted how someone else’s desires would change over time, preschool children did not. We argue that this difficulty cannot be attributed to children being biased by their current state. This pattern did not differ as a function of age suggesting that younger and older preschoolers alike have difficulty appreciating that physiological states wax and wane over time and hence that current desires may change in the future.

### 4.1. Explanatory note

We did not analyze children’s explanations because (1) 17% of children were not recorded and, therefore, their explanations could not be included in the analyses; and (2) 67% of children’s responses (of those children who were recorded) were classified as “no response/“don’t know”/irrelevant/general statement.” Because this leaves only 33% analyzable explanations, we lacked the statistical power to make valid conclusions based on these data. Future studies could include a standardized set of prompts that may draw out additional reasoning from children about their choices.
